# Adverse childhood experiences and subsequent impact on adulthood cognitive impairment: a systematic review and meta-analysis

**DOI:** 10.3389/fpsyt.2025.1751619

**Published:** 2026-01-21

**Authors:** Xin-chao Wang, Zhen-peng Huang

**Affiliations:** Faculty of Nursing, Guangxi University of Chinese Medicine, Nanning, China

**Keywords:** adults, adverse childhood experiences, childhood trauma, cognitive impairment, meta-analysis, systematic review

## Abstract

**Background:**

Cognitive impairment is a significant threat to adult quality of life. Adverse childhood experiences (ACEs) may have prolonged effects on cognitive impairment in adulthood. This systematic review and meta-analysis aimed to investigate the association between ACEs and adult-onset cognitive impairment.

**Methods:**

We systematically searched databases, including PubMed, Embase, Web of Science, and the Cochrane Library, from inception through February 16, 2025. Two independent investigators conducted literature screening, data extraction, and quality assessment using the Newcastle-Ottawa Scale. Meta-analysis was performed applying RevMan5.4.1.

**Results:**

Four studies- two cross-sectional studies, one cohort study, and one case-control study-were included. Meta-analysis results indicated that the Childhood Trauma Questionnaire (CTQ) total score (mean difference [MD]= 7.06, 95% confidence interval [CI] [0.87,13.24], *p* = 0.03), emotional neglect (MD = 2.88, 95%CI [0.39, 5.37], *p* = 0.02) were related to cognitive impairment. In the <65-year group, the MD = 2.59 (95%CI [0.09, 5.09], *p* = 0.04) was associated with cognitive impairment. The funnel diagram of gender analysis was approximately symmetrical visually, indicating a small possibility of publication bias; but due to the small number of studies, the test power was limited.

**Conclusion:**

Adverse childhood experiences may be associated with an increased risk of cognitive impairment, but a causal relationship could not be established owing to limited observational evidence.

**Systematic review registration:**

https://www.crd.york.ac.uk/PROSPERO/view/CRD420250655749, identifier CRD420250655749.

## 1 Introduction

Adverse childhood experiences (ACEs), operationally defined as exposure to potentially traumatic events occurring before the age of 18 years that pose a threat to a child’s physical or psychological safety or well-being (1, 2). Epidemiological data from the United States reveal that 58% of children experience at least one traumatic event, with notably higher prevalence rates of physical and emotional abuse (3). Another study in Brazil indicates that 34.3% of children have been exposed to trauma between 6 and 11 years of age (4). Moreover, a cross-sectional study from China suggests that 80.9% of children have been exposed to childhood trauma (5).

Recent studies have also demonstrated that such adverse experiences in early life function as a potent developmental stressor, elevating lifetime depression risk and inducing multilevel neurobiological alterations; this effect may persist into adulthood, potentially correlating with the onset and progression of cognitive impairment (6, 7). Cognitive impairment is defined as a clinically significant decline in one or more cognitive domains, namely, attention, memory, executive function, language, visuospatial skills, processing speed, or social cognition, that is severe enough to interfere with everyday activities (8). Research findings indicate that experiences of childhood trauma are associated with specific cognitive deficits in adulthood, such as difficulties in retrieving contextual memory, declines in executive function, and slowed information processing speed (9, 10). Additionally, individuals with a history of childhood trauma often exhibit reduced social adaptability when faced with complex social situations, which might be partly due to the negative effects of ACEs on brain development (11).

This study integrated international observational studies through a meta-analysis to elucidate the intrinsic link between ACEs and cognitive impairment in adulthood. Specifically, this study aimed to quantify effect sizes in terms of the association between ACEs and adult cognitive impairment, examine potential moderating effects of specific trauma-related characteristics, and assess the methodological quality of the included studies along with sources of heterogeneity.

## 2 Materials and methods

### 2.1 Study design

This systematic review and meta-analysis was guided by the Joanna Briggs Institute Manual for Evidence Synthesis and the Cochrane Handbook for Systematic Reviews. The Preferred Reporting Items for Systematic Reviews and Meta-Analyses and the Meta-analysis and Systematic Reviews of Observational Studies guidelines were used to report this systematic review. This review was registered with the International Prospective Register of Systematic Reviews (No. CRD420250655749).

### 2.2 Inclusion and exclusion criteria

The inclusion criteria were as follows: participants in the study must have clear documentation of their adverse childhood experiences exposure status, including individuals with ACEs exposure identified via assessments using scales such as the Childhood Trauma Questionnaire. Additionally, both an ACEs-exposed group and a non-exposed group should be included, including both exposed and non-exposed groups; cross-sectional studies, cohort studies, and case-control studies; and studies investigating risk factors and predictive models of cognitive impairment associated with childhood trauma. All the articles were published in Chinese or English.

The exclusion criteria were as follows: duplicate publications or data derived from the same study; studies for which full text or complete data could not be obtained; and article types such as conference abstracts, abstracts without full text, and reviews.

### 2.3 ACEs exposure criteria

The childhood trauma questionnaire (CTQ) is a commonly used tool, designed to quantify an individual’s experiences of emotional abuse, physical abuse, sexual abuse, emotional neglect, and physical neglect during childhood (12). The CTQ consists of 58 items and 5 subscales. For any subscale, a score exceeding 9 points out of the maximum 28 points is defined as a positive result, indicating the potential presence of the corresponding type of traumatic history. The ACE Scale is another commonly used instrument to assess adverse events experienced during childhood such as domestic violence and sexual abuse (13). An ACE Scale score of above 1, indicating at least one adverse event, is typically used as the threshold.

The diagnostic criteria for cognitive impairment were as follows: according to the diagnostic and statistical manual of mental disorders, fifth edition (DSM-V), cognitive impairment is diagnosed based on specific clinical symptoms and functional impairment criteria; according to the DSM-IV, the diagnosis of cognitive disorders is based on specific clinical symptoms and functional impairment criteria; the structured clinical interview for DSM (SCID) is used to assess cognitive disorders; other cognitive function scales, such as CDR, MMSE, MoCA are used for assessment and meet the diagnostic criteria for cognitive disorders in DSM-IV (14–17).

### 2.4 Search strategy

A combination of subject headings and free-text terms was used to systematically search China-based and international databases, including the PubMed, Embase, Web of Science, and Cochrane Library. The search terms in both Chinese and English included “childhood trauma”, “adverse childhood experiences”, “adult cognitive impairment”, “cognitive function”, “cognitive dysfunction”, “cognitive function assessment”, and “risk factors”. The search was conducted until February 16, 2025. The search strategy for the English databases is presented in **Supplementary Table 1**.

### 2.5 Literature screening and data extraction

Two researchers independently conducted literature screening and data extraction. Note Express was used to remove duplicates and screen the retrieved articles. Key data from the included studies were extracted and verified. Any disagreements during the screening or data extraction were resolved by consulting a third researcher. The extracted data included the first author, publication year, sample size, country, diagnostic criteria, and risk factors for cognitive impairment.

### 2.6 Quality assessment of studies

Two researchers independently assessed the quality of the studies using the Newcastle-Ottawa Scale (NOS). Discrepancies were resolved through discussion with a third researcher. The NOS evaluates three aspects: selection of study groups, comparability of groups, and measurement of outcomes or exposure, totaling 9 items with a maximum score of 9 points. Studies with scores below 3 were classified as low quality, those with scores between 4 and 6 were classified as moderate quality, and those with scores above 7 were classified as high quality.

### 2.7 Statistical methods

A meta-analysis was conducted using RevMan 5.4.1 software (18). For dichotomous variables, the odds ratio was used as the effect measure, whereas for continuous variables, the standardized mean difference (MD) and 95% confidence intervals (CIs) were employed. Heterogeneity was assessed using the I² statistic and p-values. In cases where *I*²< 50% and *p*> 0.100, indicating low or no heterogeneity, a fixed-effects model was used. Conversely, in cases where *I*²≥ 50% and *p*≤ 0.100, indicating significant heterogeneity, a random-effects model was applied. When substantial heterogeneity was present, sensitivity analyses were conducted by comparing the consistency of results between the fixed-effects and random-effects models and by using a leave-one-out approach to identify potential sources of heterogeneity.

## 3 Results

### 3.1 Literature screening results

**Figure 1:**
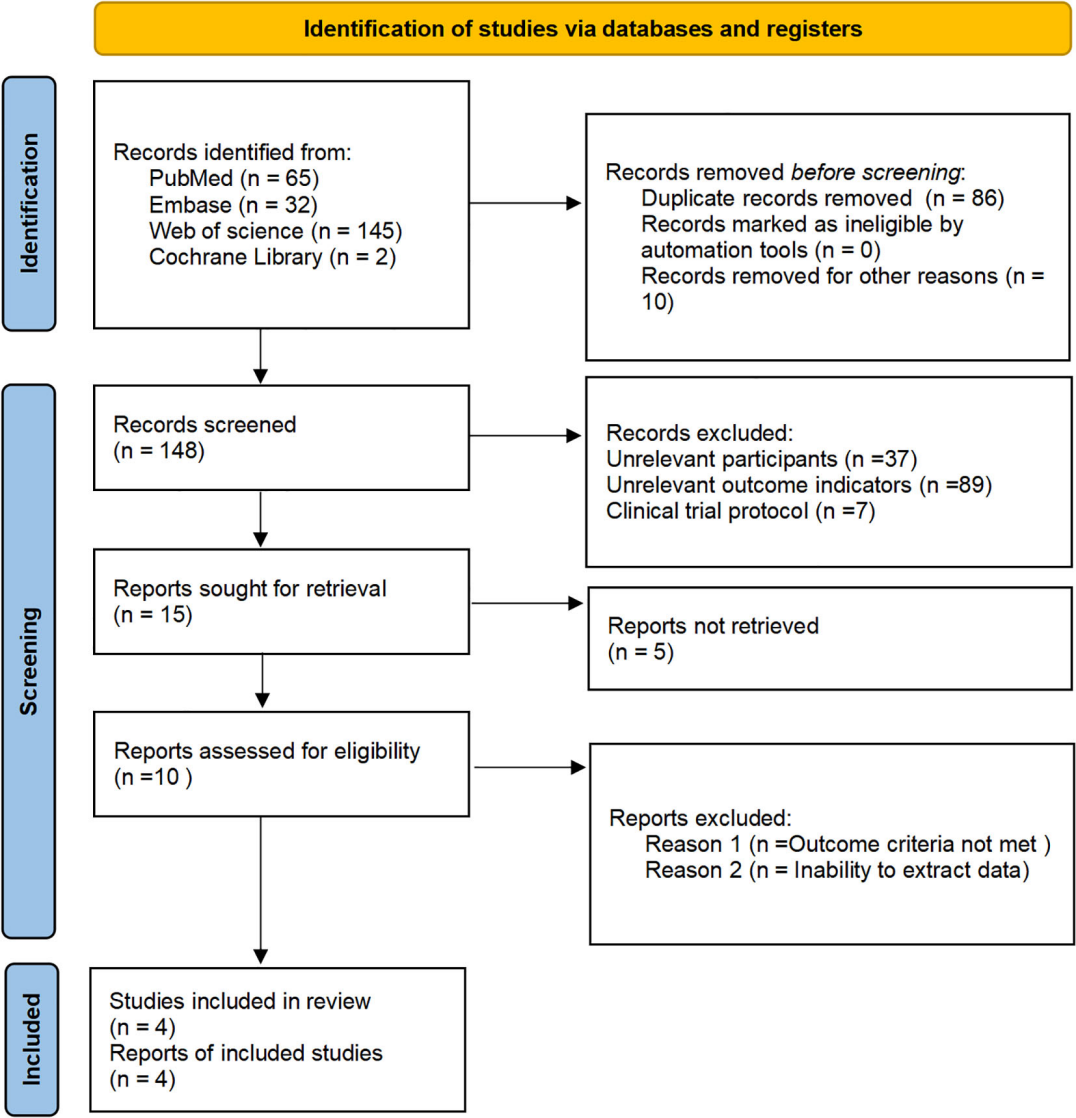
Flow diagram of literature screening between ACEs and subsequent impact on adulthood cognitive impairment.

The initial database search yielded 244 records. After removing duplicates and other ineligible items (n= 96), 148 articles remained. Title and abstract screening excluded an additional 133 articles. Finally, 15 full-text manuscripts were retrieved; however, five of these could not be obtained. Among the ten assessed for eligibility, one was excluded for unmet outcome criteria and five for unusable data, resulting in four studies being included in the final analysis. The process is set out in **Figure 1**.

### 3.2 Characteristics and quality assessment of the included studies

The total sample size in the four included studies including two cross-sectional studies, one cohort study, and one case-control study, was 1979 participants, with clear diagnostic criteria. According to the NOS assessment, all the included studies were rated as high quality. The specific characteristics and quality assessments of the included studies are presented in **Table 1** (19–22).

**Table 1:** Characteristics and quality assessment of included studies.

Included studies	Publication date	Research type	Country	Sample size (E/NE)	Age (E/NE)		Gender (Male/Female)		Diagnostic criteria	Influencing factors	NOS score
Lian J (21)	2024	Cohort Study	Australian	1568 (299/1269)	75.1 ± 1.6	75.1 ± 1.5	663/606	161/138	DSM-IV	1, 2, 3	7
Kaczmarczyk M (20)	2018	Cross-sectional Study	Germany	143 (68/75)	37.4 ± 9.3	35.1 ± 9.2	31/37	26/49	DSM-IV	1, 3, 4, 5, 6, 7, 8	8
Wang L (22)	2016	Case-Control Study	China	137 (76/61)	73.3 ± 5.6	70.5 ± 5.1	32/44	28/33	MMSE and MoCA	1, 2, 3, 4, 5, 6, 7, 8	8
Dannehl K (19)	2017	Cross-sectional Study	Germany	131 (91/40)	37.4 ± 12.4	34.3 ± 11.6	33/58	14/26	DSM-IV and SCID	1, 2, 3, 4, 5, 6, 7, 8	8

1 for Age, 2 for Gender, 3 for Childhood Trauma Questionnaire (CTQ), 4 for Emotional Abuse, 5 for Physical Abuse, 6 for Sexual Abuse, 7 for Emotional Neglect, 8 for Physical Neglect.

### 3.3 Meta-analysis results

**Figure 2:**
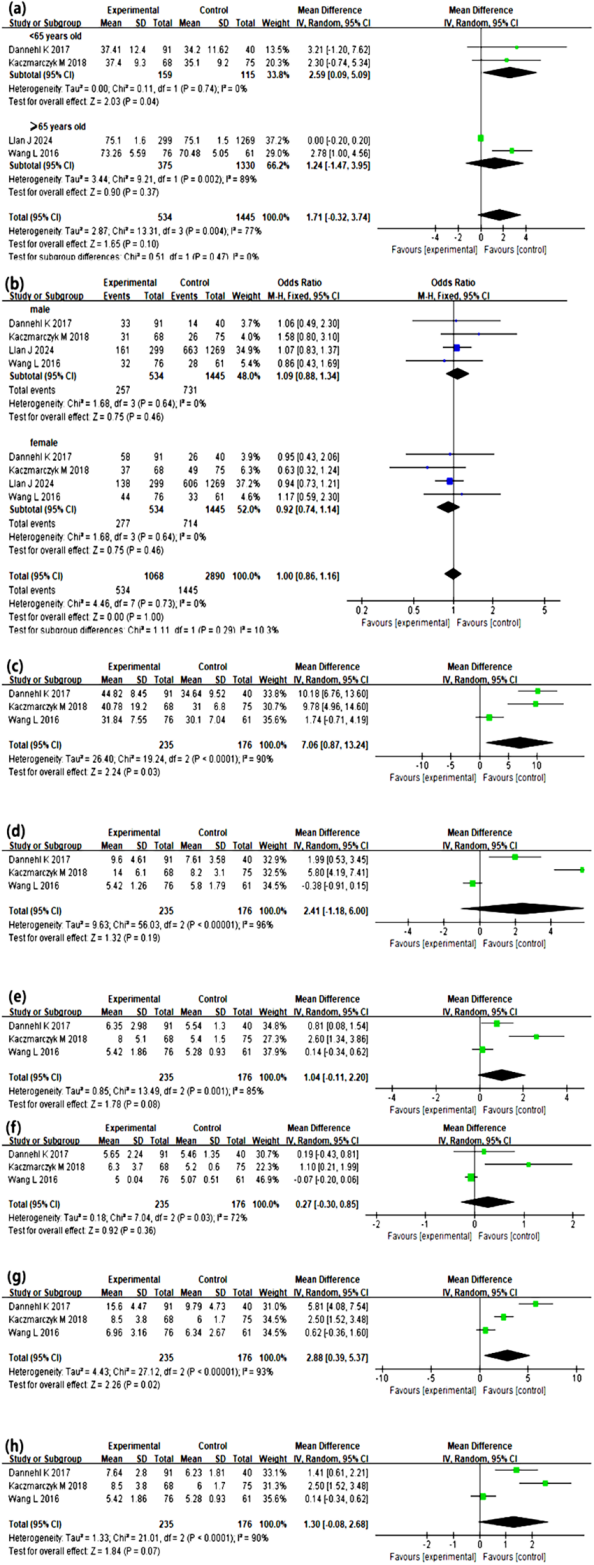
Meta-Analysis results between ACEs and subsequent impact on adulthood cognitive impairment. **(a-h)** shows the forest plot illustrating the mean differences and 95% confidence intervals of cognitive disorder group and non-cognitive disorder group on each corresponding indicator. Age **(a)**, gender **(b)**, CTQ score **(c)**, emotional abuse **(d)**, physical abuse **(e)**, sexual abuse **(f)**, emotional neglect **(g)** and physical neglect **(h)** were taken into meta-analysis. Under 65 years old, CTQ total score, emotional neglect, all were significant risk factors for cognitive impairment associated with ACEs (all *p* < 0.05).

Age, gender, the CTQ score, emotional abuse, physical abuse, sexual abuse, emotional neglect, and physical neglect were considered in the meta-analysis. Among the identified risk factors, the CTQ total score, emotional abuse, physical abuse, and physical neglect exhibited high heterogeneity, prompting further investigation into the sources of heterogeneity. The meta-analysis results indicated that the total CTQ score, emotional neglect, and being under 65 years old were significant risk factors for ACE-associated adult cognitive impairment (all *p* < 0.05). The detailed results are presented in **Table 2**, **Figure 2**.

**Table 2:** Risk factors for subsequent adult cognitive impairment impact on childhood trauma.

Item	Number of studies	Number of participants	Heterogeneity test		Results of meta-analysis			
			*I^2^*(%) *p*-value		Effect size	95% *C.I.*	Effect model	*p*-value
Age	4 (19–22)	1979	77	0.004	MD=1.71	(-0.32, 3.74)	Random	0.10
<65 years	2 (19, 20)	274	0	0.74	MD=2.59	(0.09, 5.09)	Random	0.04
≥65 years	2 (21, 22)	1705	89	0.002	MD=1.24	(-1.47, 3.95)	Random	0.37
Gender	4 (19–22)	1979	0	0.73	OR=1.00	(0.86, 1.16)	Fixed	1.00
CTQ score	3 (19, 20, 22)	411	90	<0.0001	MD=7.06	(0.87, 13.24)	Random	0.03
Emotional Abuse	3 (19, 20, 22)	411	96	<0.0001	MD=2.14	(-1.18, 6.00)	Random	0.19
Physical Abuse	3 (19, 20, 22)	411	85	0.001	MD=1.04	(-0.11, 2.20)	Random	0.08
Sexual Abuse	3 (19, 20, 22)	411	72	0.03	MD=0.27	(-0.30, 0.85)	Random	0.36
Emotional Neglect	3 (19, 20, 22)	411	93	<0.0001	MD=2.88	(0.39, 5.37)	Random	0.02
Physical Neglect	3 (19, 20, 22)	411	90	<0.0001	MD=1.30	(-0.08, 2.68)	Random	0.07

## 4 Discussion

This study found that exposure to ACEs significantly increased the risk of cognitive impairment among individuals under 65 years of age, however, this was not true for those aged 65 and older. This difference may stem from age-related physiological and psychological mechanisms evolution. In individuals under 65 years, ACEs can dysregulate the hypothalamic-pituitary-adrenal axis, leading to abnormal cortisol levels and subsequent impairment of key brain regions like the hippocampus (23). Concurrently, ACEs often induce long-term mental health issues, which further exacerbate the process of cognitive impairment (24). However, individuals aged 65 years and older, age-related physiological decline has become the dominant risk factor for cognitive impairment. The natural aging of the brain, declining organ function, and a high prevalence of chronic diseases including hypertension and diabetes, which may overshadow the effects of ACEs (25, 26). Furthermore, older survivors of ACEs may possess greater psychological resilience, developed through a lifetime of cognitive reappraisal and adaptive mechanisms, which can buffer the long-term effects of early trauma (27, 28).

Our findings showed that the CTQ total score was significantly associated with increased cognitive impairment risk, consistent with previous studies (29). ACE-related changes may exacerbate the long-term effects of ACEs on the brain, thereby increasing the risk of cognitive impairment (30, 31). ACEs and cognitive impairment in that ACEs can affect brain development and function in several ways, with increased risk of cognitive impairment (32). After facing multiple and severe ACEs, such individuals experience a notable deterioration in attention, memory, and executive function (33, 34), through mechanisms involving ongoing overactivity of the stress response system and prolonged exposure to high stress hormones, which can cause changes in brain structure, such as shrinkage of the prefrontal cortex (35).

Furthermore, no significant associations were observed between other types of ACEs assessed by the CTQ and cognitive impairment in our study. This could be attributed to the relatively high educational attainment in our study population, as higher education may provide cognitive reserve that buffers the negative impact of ACEs (36). Additionally, definitions and assessments of ACEs vary across studies. Relying solely on the CTQ may not capture the full spectrum of childhood adversities, it should explore the application of different assessment tools in this population to better elucidate the factors contributing to cognitive impairment in individuals with ACEs in future research.

In addition, our findings showed that the dimension of emotional neglect was slightly significantly associated with increased cognitive impairment risk, consistent with previous studies (37). A lack of emotional support and physical care in chronic conditions may lead to deficiencies in emotional regulation and social adaptability, thereby affecting brain development, particularly in regions associated with cognitive, executive, and emotional regulation (38). Specifically, emotional neglect may alter functional connectivity in the brain, particularly in areas related to emotional regulation and cognitive flexibility, further accelerating cognitive decline (39, 40). However, this difference only reflects findings in relation to cross-sectional data, meaning that causality and the effect of excluding confounding factors such as education and depression remain to be determined.

At the neurophysiological level, ACEs are linked to restricted blood flow to the prefrontal cortex, impeding development, which was subsequently linked to poorer executive functioning (41, 42). ACEs are also associated with elevated cortisol, promoting amyloid β plaque deposits. The accumulation of these plaques is related to brain atrophy and accelerated adulthood cognitive impairment (41). Additionally, ACE may be related to heightened levels of systemic inflammation, it also would play an important role in the pathogenesis of adulthood cognitive impairment (42).

In this meta-analysis, we observed substantial heterogeneity. This heterogeneity mainly stemmed from the following aspects. Firstly, the wide age range 37–75 years in CTQ analyses may have introduced bias due to differences in baseline health, cognitive function and recall perspectives across age groups. Secondly, two enrolled patients had major depressive disorder, which is an important confounder as MD is associated with both higher ACEs incidence and cognitive impairment (43, 44). Thirdly, common modifiable risk factors were insufficiently controlled, specifically, common modifiable risk factors, including diabetes, thyroid dysfunction, cardiovascular disease, and sensory impairments, were not systematically controlled or excluded (45–47).

This study has several limitations. First, the retrospective observational study designs utilized in the included research did not establish causal relationships but rather indicated associations. However, given the nature of our study, these designs were the most practical and suitable approach. Second, the number of included studies and the number of events may limit the generalizability of our results.

## 5 Conclusion

In conclusion, the findings of this systematic review and meta-analysis have suggested that ACEs may be associated with an increased risk of cognitive impairment in adulthood, specifically being under 65 years old, the total CTQ score and emotional neglect. The findings also indicate that early identification should be undertaken among those with cognitive impairment associated with ACEs.

## Data Availability

The original contributions presented in the study are included in the article/**Supplementary Material**. Further inquiries can be directed to the corresponding author.
