# Supplementary material for: Adverse childhood experiences and subsequent impact on adulthood cognitive impairment: a systematic review and meta-analysis
Source: Front Psychiatry. 2026 Jan 21;16:1751619. doi: 10.3389/fpsyt.2025.1751619 (PMC12869310; doi:10.3389/fpsyt.2025.1751619)
Supplement: Supplementary file 1 [file Table1.docx]

**Table1 Search strategy for the databases**

| #1 (("childhood trauma"[Title/Abstract] OR "adverse childhood experiences"[Title/Abstract]))  #2 ("adult cognitive impairment"[Title/Abstract] OR "cognitive function"[Title/Abstract] OR "cognitive dysfunction"[Title/Abstract] OR "cognitive function assessment"[Title/Abstract])  #3 #1 AND #2 |
| --- |
